# Factor Analysis and Regression Prediction Model of Swimmers' Performance Structure Based on Mixed Genetic Neural Network

**DOI:** 10.1155/2022/2052975

**Published:** 2022-05-31

**Authors:** Rui Yuan, Yuexing Han

**Affiliations:** ^1^Physical Education Department, Shanghai Maritime University, 1550 Haigang Avenue, Pudong District, Shanghai 201306, China; ^2^School of Computer Engineering and Science & Shanghai Institute for Advanced Communication and Data Science, Shanghai University, Shanghai 200444, China

## Abstract

With the development of economy, people put forward higher demands on material life and spiritual life, and sports have become an indispensable part of daily life. Ten km freestyle, although it has been started late, is loved by people all over the world, and it is getting more and more attention in the world of competitive sports. Through the theoretical research of 10 km freestyle, it can be seen that most of the research on this project is focused on the training, selection of materials, and how to improve the technique of crossing obstacles or pools, while the research on the performance of 10 km freestyle is limited to the analysis of the development trend of the performance and no research on the prediction of the actual performance, which to some extent restricts the scientific development of 10 km freestyle. The efficiency of a suitable learning is a factor that influences whether the training and learning of a BP neural network is stable or not. It can also directly determine the choice of weights. When the learning rate is chosen to be large, it increases the speed of learning and makes the stability of the network change. This has limited the scientific development of 10 km freestyle to a certain extent, and it is also a good entry point for the research of 10 km freestyle. BP neural network is one of the most widely used neural network models, which can be used for classification, clustering, prediction, and other related problems. In this study, we propose a comprehensive evaluation method of 10 km freestyle performance based on BP neural network and try to establish a neural network evaluation model by combining the athletes' physiological and biochemical indexes and sports performance. In this study, the normalized index values are used as the network input, and the athletes' performance in 10 km freestyle is used as the network output to predict the athletes' performance in 10 km freestyle. This study provides a theoretical basis for adjusting the condition of 10 km freestyle athletes and improving their performance. Optimize the reasonable allocation of resources between coaches and athletes, and increase the opportunities for coaches and athletes to communicate and learn from outside.

## 1. Introduction

With the improvement of people's living standard and material level, sports are becoming more and more important part of people's daily life, and athletics has been one of the most popular competitive groups in sports competitions and daily sports [1]. Some African countries such as Jamaica, South Africa, and Nigeria, from playing their own characteristics and geographical environment advantages through track and field sports in the middle and long distance swimming, greatly show their countries and nationalities in the international family image and enhance its status in the world sports [2]. Sports performance prediction has always been a concern for coaches, athletes, and sports researchers [3]. In recent years, the extensive use of BP neural networks has provided an effective method and a large research space for sports performance prediction research [4]. The freestyle event in track and field, especially the 10 km freestyle event, although it is late in the family of track and field, has been accepted and loved by people from all over the world over the years [5–8].

The freestyle event originated in Britain and has a history of 100 years since the 19th century. However, its development has been very tortuous, from the creation of the project to its first appearance in the Olympic Games, took more than half a century, until the 7th Summer Olympics in Belgium was included in the regular Olympic events for the first time. Then, due to the influence of the Second World War, the performance of 10 km freestyle had obvious fluctuations. 50s post-war new order was established and the world re-entered the stage of peace, the level of the steeplechase was developed by leaps and bounds, and the world record was constantly set, and now, it has exceeded the 8 minutes' mark [9]. Looking at the trend and future development of 10 km freestyle in the Olympic Games, there is still some room for the development of this sport [10]. Recent papers summarize the rules and training and competition characteristics of freestyle, which can not only improve the training and competition of athletes scientifically and effectively [11] but also provide theoretical support for training excellent athletes [12–14]. The monitoring of routine physiological indicators such as weight, blood pressure, morning pulse, and oxygen saturation, during the training and adjustment of athletes, is of certain significance for coaches to adjust training plans and develop training programs, thus helping athletes to improve their athletic performance [15]. And, if these routine physiological indices can be used for sports performance prediction, it will provide more direct and effective help for coaches and athletes in training and adjustment [16].

Quantitative sports performance prediction is a topic of interest to sports practitioners for research, decision-making, and training. The prediction of sports performance can be seen as a nonlinear mapping from athletes' performance parameters to competition performance, and BP neural network can also be seen as a nonlinear mapping from input to output. Therefore, in this study, we use the idea and algorithm of BP neural network to build an artificial neural network model for predicting athletic performance based on the regular physiological index parameters of athletes. The model was successfully established. The successful establishment of the model not only provides important scientific and technological support for coaches to reasonably arrange training and prepare for competitions but also provides important guidance on the development trend of sports performance and provides an important theoretical basis for decision makers. The thesis also focuses on the analysis of relevant physiological index factors that affect athletes' performance and gives scientific explanations and corresponding guidance. The thesis is a practical reference for the future development of various sports performance prediction studies and can be used to assist decision-making and determine the training and development goals of competitive athletes.

## 2. Analysis of Achievement Structure Based on Hybrid Genetic Neural Networks

### 2.1. Normalization of Input Data

In practical research, the units of input data and the range of values, large values, or large ranges of data tend to be overly “valued” in machine learning, while small values or small ranges of data are often “valued” by machine learning, and this phenomenon results in slow convergence and long operation times [11,17,18]. The result is slow convergence and long operation time [19]. Therefore, for data processing in neural networks, we need to preprocess the data in the initial stage of the algorithm. In this study, we mainly use the normalization method to process the original data, and the common linear normalization algorithm has two forms:

(1) $$\begin{array}{c} y=\frac{x\min}{x\max-x\min}, \end{array}$$

where *x* is the current data to be processed, min is the minimum value of the data, max is the maximum value of the data, and *y* is the normalized result. The data can be normalized to the [0, 1] space by changing the conversion of the formula:

(2) $$\begin{array}{c} y=2\frac{x\min}{x\max-x\min-1}. \end{array}$$

This formula normalizes the data to the [−1, 1] interval.

The linear transformation relationship between the input and output of neurons in this layer can be expressed as follows:

(3) $$\begin{array}{c} x_{i}=N_{i}^{l}\left(N\right),\\ y{\left(x\right)}_{i}=f_{i}\left(N_{i}^{l}\left(N\right)\right),\\ f_{i}\left(N_{i}^{l}\left(N\right)\right)={\text{net}}_{i}\left(N\right),\;i=1,2\ldots , \end{array}$$

where *x*_i_ is the input signal of the input layer and the input variables are the position synchronization errors, respectively:

(4) $$\begin{array}{c} x_{i}^{l}=\frac{e_{y1}-e_{y2}}{e\left(t\right)}. \end{array}$$

Speed synchronization error is

(5) $$\begin{array}{c} x_{1}^{l}=\frac{e_{y1}-e_{y2}}{e\left(1\right)},\\ x_{2}^{l}=\frac{e_{y1}-e_{y2}}{e\left(2\right)}. \end{array}$$

where *e*_1_ and *e*_2_ are the position tracking errors of the *Y*_1_-axis linear motor and *Y*_2_-axis linear motor, respectively, *y*_i_(*N*) is the output signal of the input layer, and *N* is the number of sampling times.

In addition, MATLAB also provides common data normalization functions, such as premnmx, postmnmx, and tramnmx, which are 3 functions [20, 21].

According to the above principles of neural network parameter setting, in this study, the neural network parameters are set as shown in Table 1.

### 2.2. Determination of Neural Network Structure

The first step in building a reliable neural network model is to determine the structure of the neural network. Based on this, the parameters are as follows: the implicit layer transfer function is tansig, the output layer transfer function is tansig, the training function is purelin, the display interval is 10, the network learning rate is 0.001, the maximum number of training is 50,000, and the target error is 0.65*∗*10^(−11). In order to prevent the phenomenon of overfitting during the experiment, which causes the network model to have low prediction performance and weak generalization ability, 3-fold cross-validation is used in this study, and all prediction results are the average value after 3 times of cross-validation.

The construction process of the neural network model requires the setting of various parameters. The appropriate parameter settings can not only ensure the accuracy of the model construction and the best prediction effect and reduce the error but also greatly reduce the computing time and determine the principle of

1. Network nodes: the number of neuron nodes in the input layer of the network is the number of feature factors of the system, and the number of neuron nodes in the output layer is the number of targets of the system. In general, the number of nodes in the hidden layer is usually set to 75% of the number of nodes in the input layer.
2. Determination of initial weights: the setting of initial weights is generally determined empirically and cannot be set to an exactly equal set of values.
3. Training rate setting: in the classical BP algorithm, the training rate is determined empirically, and the larger the training rate is, the larger the weight change will be and the convergence speed will be accelerated; if the training rate is too large, it may cause the system to oscillate. Therefore, the principle of setting the training rate is the larger the better without causing oscillations.
4. Dynamic parameters: it is usually set between 0.6 and 0.8.
5. Allowable error: generally, set to 0.001∼0.00001, when the error is less than this value, that is, the allowed error range is reached and the system ends the iteration and gives the operation result.
6. Number of iterations: since the neural network does not guarantee that the final iteration result will converge with the expectation of probability 1, the number of iterations indicates the maximum number of operations allowed when the iteration result does not converge.

Building a complete BP neural network consists of three main parts: model design, model training, and prediction simulation.

Accordingly, the main functions of MATLAB neural networks to be used in the study include.

#### 2.2.1. Newff: Feedforward Network Creation Function

The newff function can be expressed as net = newff (P, T, S, TF, BTF, BLF, PF, IPF, OPF, DDF), where P table is a certain number of input data, *T* is the output data of this feedforward network function, S is the result of each neuron, TF is the transfer function of the feedforward network, BLF is the training function that represents the model, OPF is the output function of the model, and DDF is the validation data division function generally set the first six parameters in the process of use, and the last four parameters are default values under normal circumstances.

#### 2.2.2. Train: Train a Neural Network

The syntax of the train function is [net, tr] = train(NET, *X*, *T*, *Pi*, *Ai*), where NET is the network to be trained, *X* is the input data, *T* is the output data, Pi is the input layer condition, Ai is the output layer condition, net is the trained network, and tr is the training process record. In practice, only NET, *X* and *T* need to be set, and Pi and Ai usually use the default values.

#### 2.2.3. Sim: Simulation Using the Network

The syntax of the Sim function is *Y* = sim (net, *X*), where net is the network and *X* is the *K*×*N* matrix input to the network, where K is the number of network inputs, *N* is the number of data samples, and *Y* is the output matrix *Q*×*N*, where *Q* is the number of network outputs.

**Figure 1:**
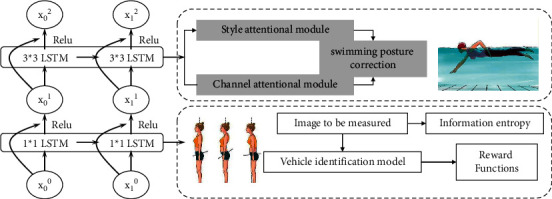
Hybrid genetic neural network model.

A hybrid genetic neural network model is constructed using MATLAB's own toolbox, as shown in Figure 1.

**Figure 2:**
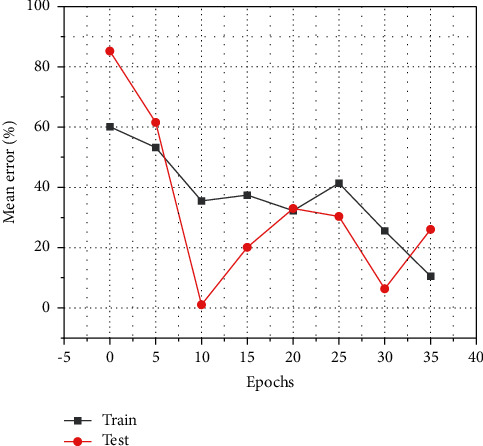
Number of neural network iterations and optimal number of iterations.

Figure 2 gives the number of 67 iterations of the BP neural network model constructed in this study, and the optimal number of iterations is the 61st one. At the 61st iteration, the MSE of the best cross-validation = 0.21714. The multiple linear regression method was done using SPSS22.0, and the BP neural network was mainly programmed using MatlabR2015b for the final prediction purpose.

However, when the learning rate is small, it makes the training time longer, the convergence slows down, and the error cannot reach the desired value. In view of the consideration of the above issues, it is considered that when choosing the learning rate, it is better to start with a small rate so that the stability of the learning convergence process can be ensured by the program written. In this study, in order to deal with the instability of existing performance evaluation methods, a model based on artificial neural network for obstacle swimming sports performance estimation is proposed, followed by a neural network evaluation model through the physiological and biochemical indexes and sports performance of obstacle swimming athletes of Ningxia sports team.

The expected error is the best error value obtained based on the comparison of each error value obtained from the training learning of the network. The number of nodes in the hidden layer directly affects the selection of the error value, and when the number of nodes in the hidden layer selected increases, the more the while error value at this time is close to the expected error value. In this study, we found that when there are 13 nodes in the hidden layer, the error value reaches the expected error value. The best expected error is 0.008. Eighty thousand training cycles are chosen.

### 2.3. Multiple Linear Regression versus Neural Network Prediction

In this study, the freestyle performance was predicted using BP neural network and multiple linear regression, respectively, with the aim of comparing the two models and specifying which one can be better used for performance prediction. Both predictions were made using the results of 33 out of 42 male freestyle swimmers and the data of four sensitive factors obtained through factor analysis. The multiple linear regression method was done using SPSS22.0, and the BP neural network was mainly programmed using MatlabR2015b for the final prediction purpose. Then, the four sensitive indicators of the remaining nine randomly selected male athletes were written into the program to complete the validation of the model. The multiple linear regression, in which the results of each index were substituted into equation *Y* = −0.446*X*1−0.095*X*2−0.895*X*3−3.288*X*4 + 136.142, where *X*1 represents the number of 1 min fast side stirrups imitations, *X*2 represents lung capacity/body weight, *X*3 represents (knee circumference + ankle circumference)/height, and *X*4 standing long jump. The predicted results were obtained by inverse normalization.  Table 2 shows the comparison between the results obtained from the multiple linear regression and BP neural network methods and the actual test values of the athletes.

**Figure 3:**
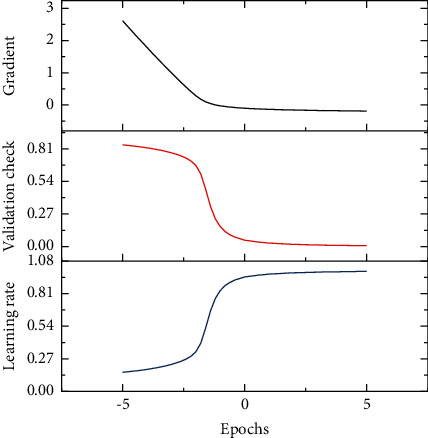
Swimming performance comparison chart.

The corresponding comparison plots are given in Figure 3, where blue is the true value and red is the predicted value. The above results show that the neural network model constructed in this study can better reflect the relationship between the quality training of 10 km freestyle athletes and the performance of 10 km freestyle and thus can provide effective support for athletes and coaches in actual training and competition.

## 3. Results and Analysis

### 3.1. Prediction of Each Parameter

**Figure 4:**
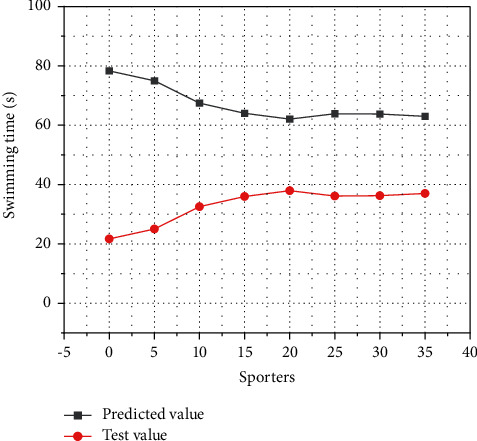
Comparison of predicted and true values of the neural network model.

Neural networks, as one of the most widely used neural network models, can be used in the research of classification, clustering, prediction, and other related problems. Currently, BP neural networks have been used very successfully in athletic status, sports performance prediction, special performance prediction of chain ball and shot put, and comprehensive evaluation of athletes' sprinting ability. And the normalized indexes are used as input, and the output is the athletic performance. Finally, the predicted values and the real values of the steeplechase runners using the artificial neural network model were compared. The analysis of the results showed that the predicted and true values obtained by using artificial neural network were similar (as shown in Figure 4), thus achieving the prediction of athletes' performance, which is an important reference value for further adjusting athletes' condition and coaches' scientific arrangement of training, adjustment, and competition to improve athletic performance. At the same time, as the athlete's oxygen saturation gradually improves, his or her performance will also improve accordingly. This is a very important reference for athletes and coaches to arrange training, adjustment, and competition scientifically.

**Figure 5:**
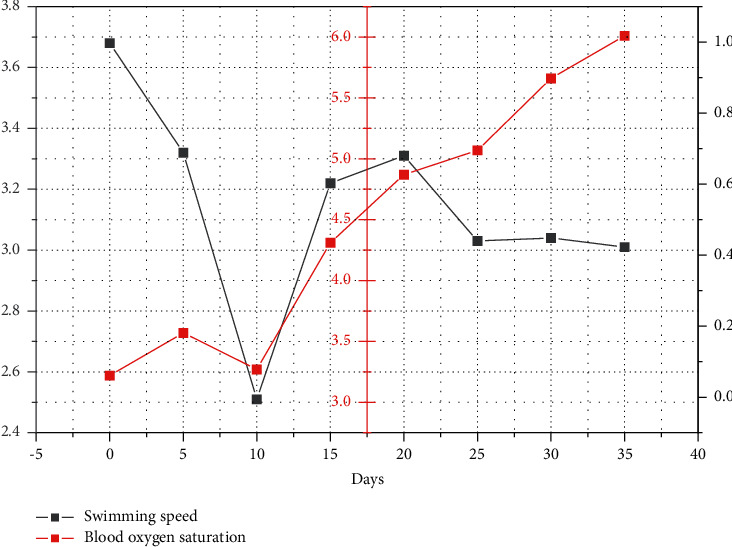
Relationship between performance and blood oxygen saturation.

In this study, we also specifically analyzed the relationship between performance and blood oxygen saturation. The analysis of the results showed a strong correlation between the two (as shown in Figure 5). In medicine, oxygen saturation is generally considered to be a normal value that should not fall below 94%, so below 94%, we consider this state as insufficient oxygenation. There is also a group of researchers who consider a state of hypoxemia when the oxygen saturation is 90%. And they believe that the accuracy can reach ±2% when the oxygen saturation is greater than 70%. It is also believed that when the oxygen saturation is below 70%, such a condition is subject to some error.

In clinical practice, oxygen saturation readings are used to directly reflect the respiratory function of the body. In practice, we have found that when athletes have consistently high oxygen saturation levels, they tend to have higher performance. When the athlete's oxygen saturation decreases significantly, there will be a significant decline in performance, which is similar to the degree of decline in oxygen saturation.

**Figure 6:**
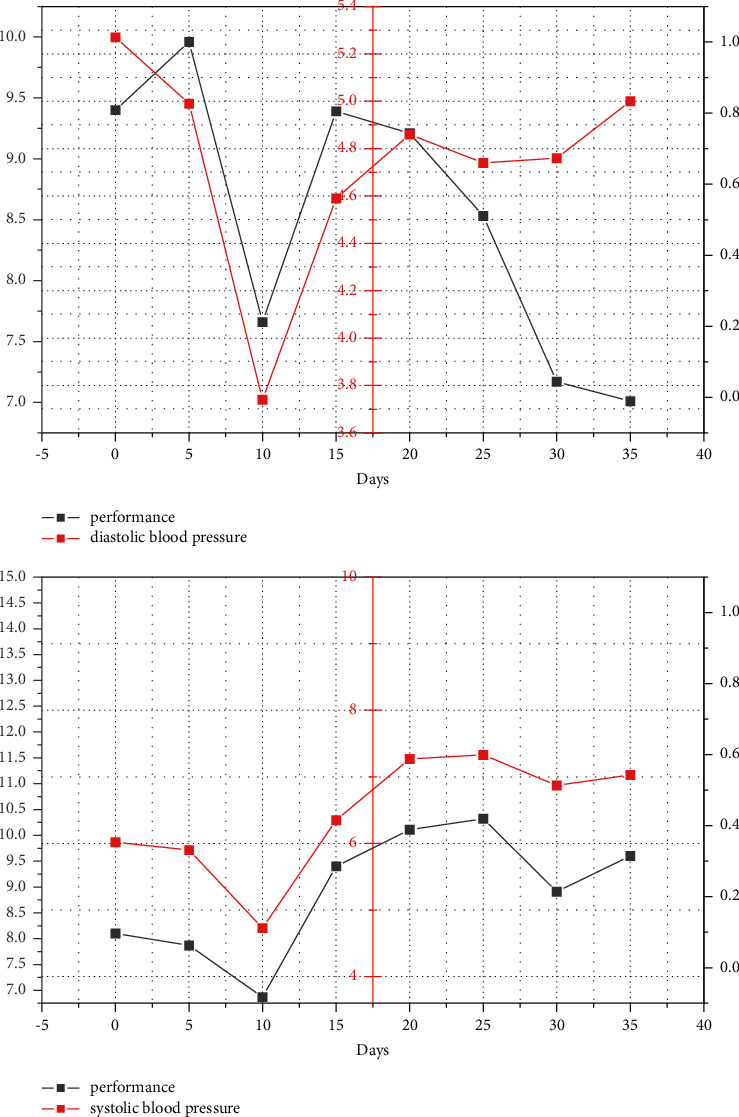
Relationship between performance and systolic and diastolic blood pressure.

As shown in Figure 6, the systolic and diastolic blood pressure of the athletes, to some extent, correlate with higher performance. At this point, then adjust it; it will take longer, thus affecting the normal training of athletes. After adjustment, systolic and diastolic blood pressure returns to normal. Further studies have shown that the physical state is better when the systolic and diastolic differential is at a critical level.

**Figure 7:**
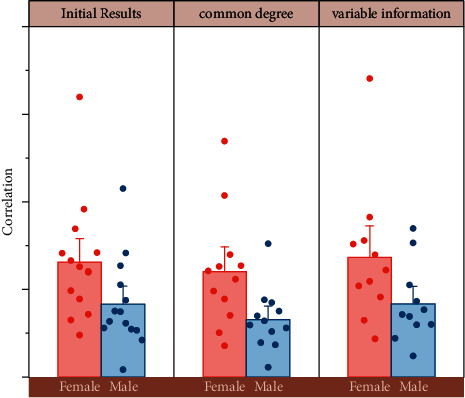
Data correlation analysis.

Figure 7 is the original 18 indicators data for principal component analysis, 18 indicators were completely extracted, and the value of the common degree of the variables in the table are 1, which means that all the variables in the original variables can be explained, and the data in the second column can be found that all the variables have a high correlation, and at this time, all the variable information is only less lost. Only by accurately grasping the characteristics of the sport, fully considering the relationship between physiological and biochemical indicators and aerobic, anaerobic and mixed training, and arranging the contents of aerobic, anaerobic and mixed training in a systematic and regular manner can the trainee's special performance be effectively improved.

Therefore, the results of this factor extraction are more satisfactory. When the principal component extraction method was adopted to extract the factors, there were only 3 factors with the value of the characteristic root greater than 1. However, the three factors could only explain 76.433% of the total variance of the original variables, which obviously could not reach 85%, so the method of extracting one more factor was chosen to make the total variance increase. When four factors are extracted, the total variance value is 81.13% still not 85%, but the later eigenvalues are small, so there is no practical meaning to extract more.

Comparing the technical and tactical skills of the best domestic and foreign swimmers, it was found that the swimmers lagged behind the best foreign female speed swimmers in terms of technical and tactical skills, ability to accelerate, and understanding and mastery of the backstroke speed reduction. This analysis refreshes the previous concept of technique and tactics in swimming and helps coaches to use training techniques flexibly, thus helping female swimmers to improve their competitive ability. This study's expert interview method, measurement method, and mathematical statistics to classify the 18 indicators with significant influence on special performance were initially formulated, using the factor analysis method to classify such indicators, so as to identify 4 representative sensitive indicators. The prediction of the model was carried out.

By analyzing the characteristics of special training loads for freestyle swimmers in China, developing speed endurance training methods, and bending technique training methods, strength training methods, and muscle group training methods, it is found that swimming is a kind of competitive sport, so to better complete the project, it is necessary to have a high level of technical and tactical skills.

**Figure 8:**
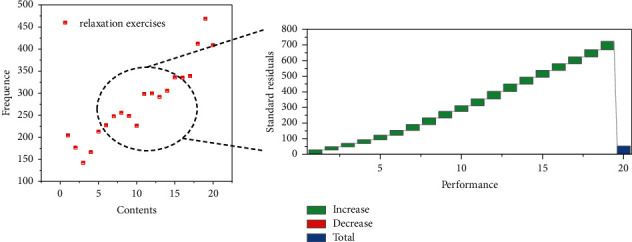
Regression standard residuals.

The study found that the correct allocation of training and relaxation practice time is particularly important for speed swimmer to improve their performance, as shown in Figure 8. Therefore, swimmers choose the appropriate relaxation exercises for their own training, which will definitely increase their performance.

Traditionally, the methods of the multiple linear regression and gray model are mainly used for prediction of special performance. By selecting factors closely related to special performance, the prediction model between special performance and sensitive indexes is established to complete the estimation of special performance. The prediction method of the multiple linear regression or gray model can reflect the correlation between sports performance and training indexes and physical quality indexes under certain circumstances and lay the theoretical foundation for coaches to accurately select talented athletes and later practice training. By comparing the two prediction methods, we found that the neural network can more accurately complete the prediction of special performance.

However, these models fitted are based on certain assumptions, which need to be assumed in advance for mathematical model formulas, such as multiple linear regression equations or power equations. The functional relationship between athletes' sports performance and selected sensitive factors is not necessarily the functional model we presuppose, so it will inevitably bring a large error to the predicted performance and lead to the irrationality of the generated results. In this thesis, we mainly used the questionnaire survey method, expert interview method, measurement method, and mathematical statistics to classify the 18 indicators with significant influence on special performance that were initially formulated, using the factor analysis method to classify such indicators, so as to identify 4 representative sensitive indicators. The prediction of the model was carried out.

Once the multiple linear regression model predictions were completed, the aim was to find a more accurate model without having to determine the mathematical expressions in advance.

## 4. Conclusion

In this study, a BP neural network model with 4 neurons in the input layer, 1 neuron in the output layer, 1 layer in the hidden layer, and 13 nodes in the hidden layer was constructed using Matlab R2015b software. Based on the two models, it was found that men's 10 km freestyle swimming performance was inversely proportional to lung capacity/body weight. Standing long jump, i.e., the greater the above four items, the less time was used for swimming, so the better the performance, the better the athlete. The network model was used to estimate the men's swimming performance more efficiently, accurately, and economically. Although both multiple linear regression and neural network can predict athletic performance better, they both have their own shortcomings in the process of prediction, so by establishing a combination of multiple models for prediction, they can play the role of complementing the shortcomings and completing the prediction of complex data. Optimize the reasonable allocation of resources between coaches and athletes, and increase the opportunities for coaches and athletes to communicate and learn from outside. Pay attention to the selection and training of men's freestyle swimmers, and apply scientific selection methods to the selection process to make the selection more targeted; apply nonspecific training methods with high relevance to the training to make the training methods more diversified. In the future, as the athlete's oxygen saturation gradually improves, his or her performance will also improve accordingly.

## Figures and Tables

**Table 1:** Setting of training parameters.

Neural network settings	Parameters	Feature dimension
Implicit layer transfer function	Tansig	2
Output layer transfer functions	Tansig	4
Training functions	Purelin	2
Display interval	10	4
E-learning rate	0.001	5
Maximum number of training sessions	5000	7
Target error	0.65*∗*10^(−11)	6

**Table 2:** Comparison of training predictions.

True value	Multiple linear regression prediction	Hybrid genetic neural network prediction
1	1.52	0.06
1	0.04	0.36
1	0.09	0.40
2	8.81	4.48
1	6.42	1.39
1	0.09	1.18
1	1.04	0.06

## Data Availability

The data used to support the findings of this study are available from the corresponding author upon request.
